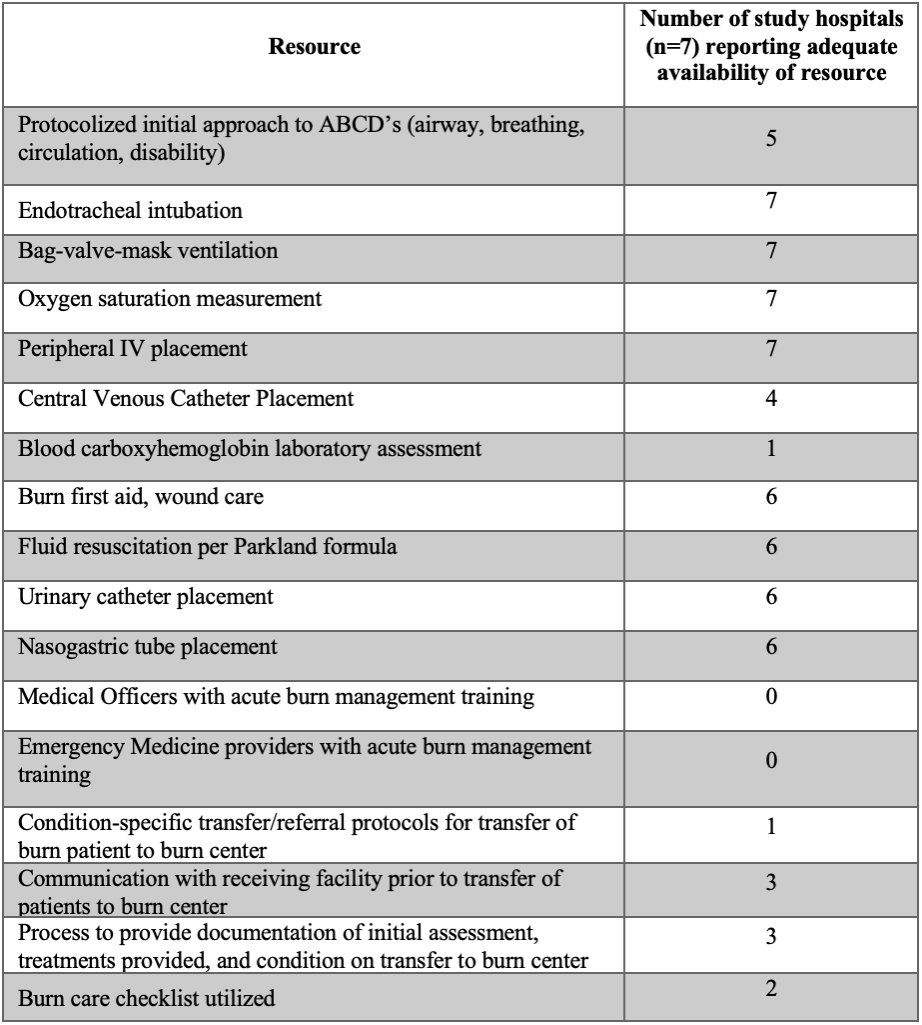# 650 Pilot Assessment of Readiness for Emergency Burn Care Utilizing a Modified W.H.O. HEAT Tool

**DOI:** 10.1093/jbcr/iraf019.279

**Published:** 2025-04-01

**Authors:** Kajal Mehta, Sharmeen Jaffry, Junaid Razzak, Manish Yadav, Barclay Stewart, Tam Pham, Adam Aluisio, Ramu Kharel

**Affiliations:** University of Washington; Brown University Warren Alpert Medical School Department of Emergency Medicine; Weill Cornell Medicine; Kirtipur Hospital; University of Washington; University of Washington; Brown University; Brown University

## Abstract

**Introduction:**

Burns are a leading cause of injury in Nepal. The 2021 Emergency Care System Assessment performed in Nepal identified several steps to improve care and facility-specific goals, which included emergency burn care. A pilot study using the World Health Organization’s (WHO) newly deployed Hospital Emergency Unit Assessment Tool (HEAT) evaluated emergency care capacity at seven tertiary hospitals in Kathmandu, Nepal. We conducted a secondary analysis of the results focused on emergency burn care capacity.

**Methods:**

This cross-sectional mixed-method pilot study utilized a modified HEAT tool to identify and describe the emergency care resources available at the study hospitals, utilizing open-ended questions, numbered responses, and discrete answers that can be used to characterize signal functions of a given facility. An expert panel modified the HEAT tool to include questions with a specific focus on “emergency burn care capacity.” Descriptive statistics and comparative analysis of emergency burn care capacity was conducted in this study.

**Results:**

Across all sites (n=7), an average 5.42/8 burn-related interventions were adequately available. Results are reported in Table 1. Five of 7 sites reported protocols in place for initial approach to ABCs (airway, breathing, circulation). All sites reported adequate airway management resources and peripheral intravenous access capabilities. Four sites reported adequate availability of central venous access. One site reported availability of blood carboxyhemoglobin laboratory assessment. No sites had medical officers or emergency medicine providers with training in acute burn stabilization. Six of 7 sites reported adequate ability to perform burn first aid, administer fluid per Parkland formula, and place urinary catheter and nasogastric tubes. One site reported having referral protocols for transfer of patients to a burn center, and 3 sites reported pre-transfer communication and provision of documentation of initial assessment and treatments with the referral burn center. Only one site reported routinely completing initial 24-hour resuscitation prior to initiating transfer. At the time of the study, 2 of 7 sites report utilizing a burn care checklist.

**Conclusions:**

The HEAT tool is a feasible methodology for assessing strengths and gaps in emergency care delivery systems, and the additional of burn care related questions provides critical information burn care system development. Integration of burn focused questions in the HEAT tool was feasible in the Nepal’s context. The assessment revealed several gaps in care including specific laboratory assessments, training in acute burn stabilization and utilization of protocols for provision of burn care and coordination of transfer.

**Applicability of Research to Practice:**

We recommend further validation of the modified HEAT tool in application to burn care systems to inform development of emergency burn care capacity in low-resource settings globally.

**Funding for the Study:**

Funding provided University Foundation Funding.